# Stage Management at a Hospital

**Published:** 1911-04-01

**Authors:** 


### STAGE MANAGEMENT AT A HOSPITAL.

BY ANOTHER HOSPITAL SECRETARY.

The article under the above heading, which was recently published in The Hospital, draws attention to an aspect of the work of our voluntary hospitals which deserves consideration by all who are responsible for their administration. Every hospital manager and secretary realises the importance of keeping the work and needs of their own institution well before the public, and they experience the difficulty of solving the problem how this publicity is to be obtained in the most effective manner and with the least expenditure of their funds.

It must be borne in mind in considering this question that the numbers of charitably-disposed persons, who are the main financial supporters of the voluntary hospitals of London, are very small when compared with the total population of the metropolitan area. It has been calculated that out of the nearly seven million inhabitants of Greater London only some twenty thousand persons systematically contribute towards the support of these institutions, which are by their necessities compelled to compete, not only with each other, but also with numerous other charitable agencies, for the support of this comparatively small section of the community. It is evident from the returns compiled and issued annually in "Burdett's Hospital Annual" and similar publications, that the amount of money which is given each year for hospital purposes does not vary much, and it is therefore evident that when any particular institution makes a specially strong and successful appeal, it does not add materially to the total contributors, but rather tends to draw to itself a large proportion of the general charitable fund which has been subscribed during the year. Every secretary of a voluntary hospital knows that it is almost useless to issue an appeal when another similar institution has already gained the ear of the public. During the past few years many of the smaller hospitals have vainly attempted to raise special funds, in face of similar efforts which have been made by their larger and more influentially-supported rivals. Of late years all the larger and old-established hospitals, including the endowed hospitals, have appealed for and obtained large sums either for extension or for maintenance.

In these circumstances the ingenuity of the secretaries and managers of the various hospitals have been taxed to the utmost to find new methods of attracting public attention to their needs. The days are past when the issue of so many thousand printed appeals or advertisements would ensure a fair return upon the outlay, for it is well known that many of such attempts to obtain funds have in recent years proved perfectly futile.

The writer of the article already referred to advocates the employment of a special publicity agent for the purpose of keeping the name of a particular hospital constantly before the public. I would, however, venture to point out that while this method of obtaining free notices in the Press may possibly be suitable for a particular kind of institution, whose class of work offers special opportunity for emotional or sensational appeals, it would soon defeat its own ends if largely adopted by many of the hundred and thirty competing hospitals of the metropolis. It is much easier for the special hospitals to obtain this kind of notice in the Press than it is for the general hospitals, who, after all, are really doing the best work for the sick poor and for medical science and education. Newspaper editors know only too well what their readers want, and newspaper proprietors and business managers are not going to give free advertisement to institutions which at the present time pay many thousands of pounds each year for advertising their appeals.

While recognising that the suggestions made by "A Hospital Secretary" well merit consideration, my fear is that it will stimulate so many of my secretarial colleagues to prepare special tit-bits relating to their hospital for the Press that the editors will soon decline to let their columns be thus utilised for the purposes of free advertisement.

